# *Paranannizziopsis* spp. Infection in Wild Vipers, Europe

**DOI:** 10.3201/eid3005.231317

**Published:** 2024-05

**Authors:** Gaëlle Blanvillain, Fernando Martínez-Freiría, Joseph R. Hoyt, Jeffrey M. Lorch, Albert Martinez-Silvestre

**Affiliations:** Virginia Polytechnic Institute and State University, Blacksburg, Virginia, USA (G. Blanvillain, J.R. Hoyt);; Centro de Investigação em Biodiversidade e Recursos Genéticos da Universidade do Porto, Vairão, Portugal (F. Martínez-Freiría);; US Geological Survey, Madison, Wisconsin, USA (J.M. Lorch);; Catalonian Reptiles and Amphibians Rescue Center, Masquefa, Spain (A. Martinez-Silvestre)

**Keywords:** fungi, Paranannizziopsis, fungal pathogen, emerging infectious disease, Iberian Peninsula, reptile dermatosis, viper, Vipera seoanei, Spain

## Abstract

We describe the detection of *Paranannizziopsis* sp. fungus in a wild population of vipers in Europe. Fungal infections were severe, and 1 animal likely died from infection. Surveillance efforts are needed to better understand the threat of this pathogen to snake conservation.

Over the past few decades, fungal pathogens have been implicated in wildlife population declines, posing a substantial challenge to the conservation of many species, including herpetofauna (*1*). In reptiles, most fungal pathogens are within the genera *Nannizziopsis*, *Paranannizziopsi*s, and *Ophidiomyces*, members of the order Onygenales (*2*). Of those genera, the most well-documented genus in wild reptiles is *Ophidiomyces*, consisting of the single species *O. ophidiicola*, which is responsible for ophidiomycosis, also called snake fungal disease (SFD) (*3*). Infections with *Paranannizziopsis* spp. fungi, on the other hand, are not well documented, possibly because of wide overlap with ophidiomycosis in how the disease manifests (*4*). Disease associated with *Paranannizziopsis* infection has been described in captive collections in North America (*2*,*5*–*7*) and Australasia (*2*,*8*). In wild populations, *Paranannizziopsis* spp. fungi have only been detected in nonnative free-living panther chameleons (*Furcifer pardalis*) from central Florida, USA (*9*), and in wild snakes in the United States and Canada (*4*). The geographic extent in wild host populations and severity of infection associated with *Paranannizziopsis* spp. fungi is unknown and deserves more thorough evaluation. We report infection with a *Paranannizziopsis* sp. fungus in 2 wild Seoane’s vipers (*Vipera seoanei*) from northwestern Spain. Handling of snakes was reviewed and approved by Virginia Tech Institute for Animal Care and Use Committee protocol 20-055. Vipers and tissue samples were collected under permit from Xunta de Galicia, Spain (permit no. EB-015/2021).

## The Study

On May 14, 2021, two *V. seoanei* vipers, a subadult male (body length 31.7 cm, weight 10.3 g) and an adult female (body length 44.7 cm, weight 61 g), were captured near Zamáns in Vigo, Spain (42.16N, 8.68W; WGS1984). Both animals were in the process of molting and displayed many skin lesions on the head and body. The lesions were particularly abundant for the subadult male, for which the molting process was abnormal (i.e., dysecdysis). The animal was lethargic and appeared moribund. This snake was brought into captivity for supportive care but died the next day. The carcass was placed in ethanol until we performed necropsy and histopathological analyses. The adult female was reproductive, and, after we collected biometric data and skin swab samples, she was immediately released at the place of capture.

We swabbed the ventral and dorsal areas of the snakes in duplicate using a premoistened, sterile polyester-tipped applicator (Puritan, https://www.puritanmedproducts.com) and stored frozen swab samples at −20°C until analysis. We extracted DNA from the samples using PrepMan Ultra Sample Preparation Reagent (ThermoFisher Scientific, https://www.thermofisher.com). In addition, we excised 7 skin lesions (≈2 × 4 mm) from the subadult male at various locations across the body and stored them in 70% ethanol. We extracted DNA using a QIAGEN Blood and Tissue kit (QIAGEN, https://www.qiagen.com) following manufacturer’s instructions, which included a lyticase lysis step (200 U for 30 min at 30°C) to degrade fungal cell walls.

**Figure 1:**
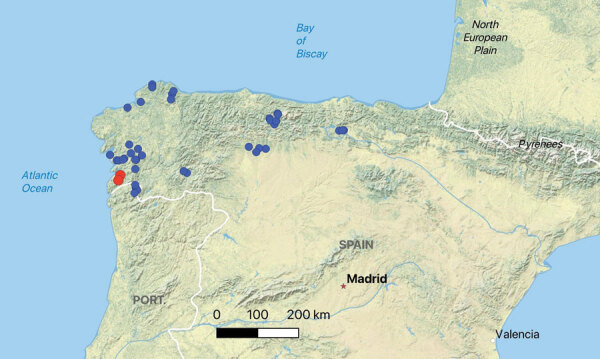
Spatial distribution of Seoane’s viper (*Vipera seoanei*) captures and detections of *Paranannizziopsis* sp. fungus in Spain and Portugal. Each dot represents an individual snake capture; overlapping points were slightly jittered for visualization. Blue dots represent snakes that tested negative by real-time PCR, and red dots represent snakes that tested positive by real-time PCR.

We screened extracted DNA from both the swab and tissue samples for the presence of *Paranannizziopsis* spp. and *O. ophidiicola* fungi using real-time PCR. *O. ophidiicola* fungi were not detected in any of the samples using a quantitative PCR (qPCR) targeting the internal transcribed spacer (ITS) region of the fungus (*10*). Samples from both vipers were qPCR-positive for *Paranannizziopsis* sp. fungus by genus-specific qPCR (*4*). We amplified and sequenced the full-length ITS and a portion of the mitochondrial cytochrome oxidase subunit III (*COX3*) gene (GenBank accession nos. OR353533 and OR351968) of the *Paranannizziopsis* sp. fungus, according to published methods (*4*). We compared sequences from those loci with existing sequences in GenBank using BLASTn (*11*). The ITS sequence most closely matched *P. australasiensis* strains in GenBank: 99.1%–99.6% identity over the ≈500 bp region sequenced for strains UAMH 12461 (OR100710), NWHC 24878–7 (OR100711), UAMH 12464 (OR100712), UAMH 12463 (OR100713), UAMH 10439 (KF477218), and UAMH 11645 (NR_111879). The *COX3* sequence shared 99.9%–100.0% identity (over the ≈670-bp portion sequenced) with sequence data from *P. australasiensis* (strain UAMH 12461 [OR103159], NWHC 24878–7 [OR103160], UAMH 12464 [OR103161], UAMH 12463 [OR103162], UAMH 10439 [OR103163], UAMH 11645 [OR164]), *P. californiensis* (strain UAMH 10693 [OR103165]), and *P. tardicrescens* (strain CBS 142038 [OR103166]) in GenBank. After positive detections for *Paranannizziopsis* sp. fungi from those 2 animals, we also screened additional skin swab samples collected from *V. seoanei* vipers in Spain and Portugal in 2020 and 2021 (n = 37, including 1 with a ventral skin lesion). We did not identify *Paranannizziopsis* spp. fungi in those additional samples, indicating a pathogen prevalence of ≈5% (2 of 39 samples) (Figure 1).

**Figure 2:**
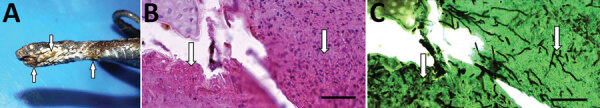
Seoane’s viper (*Vipera seoanei*) collected in Spain that was infected with *Paranannizziopsis* sp. fungus. A) Gross lesions in the mouth, on the lower jaw, and on the ventral areas of the body (arrows). B) Lightly stained hyphae (arrows)in section of epidermis stained with hematoxylin and eosin. Scale bar indicates 20 μm. C) Intralesional hyphae (arrows) in section of epidermis stained with the Grocott-Gomori methenamine silver method. Scale bar indicates 20 μm.

At necropsy, the subadult male viper had variable numbers of multifocal to coalescing, raised, white-gray to dark brown discolored cutaneous lesions, ranging in size from 1 to 6 mm in diameter, along the left side of the mouth and labial scales; the lower jaw; and the central, dorsal, and caudal regions (Figure 2, panel A). We took samples of skin, bone, stomach, liver, kidney, and intestine for histopathological analysis. Tissue sections were stained by hematoxylin and eosin and Grocott-Gomori methenamine silver stains. Microscopically, skin lesions included areas of necrosis with granulocytic inflammation in the superficial to mid-epidermis; we observed slight edema adjacent to the mid-epidermis. Small chronic inflammatory cell aggregates composed of degenerated heterophils mixed with necrotic cellular debris and proteinaceous fluid were observed in these lesions. We detected nonpigmented fungal hyphae at the epidermal surface and breaching the epidermis under hematoxylin and eosin stain (Figure 2, panel B). We observed structures morphologically compatible with hyphae under Grocott-Gomori methenamine silver stain (Figure 2, panel C). Those hyphae were 1.8–4.9 µm in diameter, were septate, and had parallel walls with irregular dichotomous branching. We did not observe any relevant lesions or fungal elements in the internal tissues and viscera.

## Conclusions

The effects of fungal diseases on reptiles have been difficult to evaluate, especially in cryptic species such as snakes. We report detection of a *Paranannizziopsis* sp. fungal infection in a wild population of *V. seoanei* vipers in Spain, and at least 1 viper likely died because of the infection. The pathology and fungal morphology were consistent with *Paranannizziopsis* spp. infections reported elsewhere (*2*,*4*–*7*). Although the strain detected in the snake that died was most similar to *P. australasiensis*, we were unable to identify the strain to a particular species of *Paranannizziopsis* fungus. Additional genetic analyses on the *Paranannizziopsis* sp. fungus detected in Spain might help better resolve its taxonomy.

Whether *Paranannizziopsis* spp. fungi are native to the Iberian Peninsula or whether our detections could represent recent transmission events from captive snakes remains unclear. We did not detect *Paranannizziopsis* spp. fungi in additional snakes sampled from Spain and Portugal, and *O. ophidiicola* fungus has not been detected in the Iberian Peninsula (*11*). Thus, if the pathogen was recently introduced, spread of this fungus to other vulnerable reptile populations is of concern, and further investigation is warranted considering the conservation need for most reptiles worldwide (*12*). *V. seoanei* vipers are a nearly endemic species to the Iberian Peninsula and is restricted to the northern region of the Atlantic climate (*13*). Populations have been severely impacted by habitat loss and fragmentation, and ecological models indicate high vulnerability of this species to climate change (*14*). Western populations, where these 2 infected snakes were found, are at the edge of the species’ distribution and are the most genetically diverse and isolated, highlighting their importance for maintaining genetic diversity (*14*,*15*). In light of this factor, detection of a species of *Paranannizziopsis* fungus raises concerns regarding the additive effects of other stressors and disease on the health of this imperiled population, and increased surveillance for this pathogen in wild populations might be warranted.
